# Effectiveness of the HoloLens mixed-reality headset in minimally invasive surgery: a simulation-based feasibility study

**DOI:** 10.1007/s00464-019-06862-3

**Published:** 2019-06-18

**Authors:** Hasaneen Fathy Al Janabi, Abdullatif Aydin, Sharanya Palaneer, Nicola Macchione, Ahmed Al-Jabir, Muhammad Shamim Khan, Prokar Dasgupta, Kamran Ahmed

**Affiliations:** 1 MRC Centre for Transplantation, Guy’s Hospital, King’s College London, 5th Floor Southwark Wing, London, SE1 9RT UK; 2 ASST Santi Paolo e Carlo, Università degli Studi di Milano, Milan, Italy; 3 Department of Urology, Guy’s and St, Thomas’ NHS Foundation Trust, London, UK; 4 Department of Urology, King’s College Hospital NHS Foundation Trust, London, UK

**Keywords:** Augmented reality, Virtual reality, Head-mounted displays, HoloLens, Endoscopy, Surgery

## Abstract

**Background:**

The advent of Virtual Reality technologies presents new opportunities for enhancing current surgical practice. Studies suggest that current techniques in endoscopic surgery are prone to disturbance of a surgeon’s visual-motor axis, influencing performance, ergonomics and iatrogenic injury rates. The Microsoft^®^ HoloLens is a novel head-mounted display that has not been explored within surgical innovation research. This study aims to evaluate the HoloLens as a potential alternative to conventional monitors in endoscopic surgery.

**Materials and methods:**

This prospective, observational and comparative study recruited 72 participants consisting of novices (*n* = 28), intermediate-level (*n* = 24) and experts (*n* = 20). Participants performed ureteroscopy, within an inflatable operating environment, using a validated training model and the HoloLens mixed-reality device as a monitor. Novices also completed the assigned task using conventional monitors; whilst the experienced groups did not, due to their extensive familiarity. Outcome measures were procedural completion time and performance evaluation (OSATS) score. A final evaluation survey was distributed amongst all participants.

**Results:**

The HoloLens facilitated improved outcomes for procedural times (absolute difference, − 73 s; 95% CI − 115 to − 30; *P* = 0.0011) and OSAT scores (absolute difference, 4.1 points; 95% CI 2.9–5.3; *P* < 0.0001) compared to conventional monitors. Feedback evaluation demonstrated 97% of participants agreed or strongly agreed that the HoloLens will have a role in surgical education (mean rating, 4.6 of 5; 95% CI 4.5–4.8). Furthermore, 95% of participants agreed or strongly agreed that the HoloLens is feasible to introduce clinically and will have a role within surgery (mean rating, 4.4 of 5; 95% CI 4.2–4.5).

**Conclusion:**

This study demonstrates that the device facilitated improved outcomes of performance in novices and was widely accepted as a surgical visual aid by all groups. The HoloLens represents a feasible alternative to the conventional setup, possibly by aligning the surgeon’s visual-motor axis.

The Microsoft^®^ HoloLens is a mixed-reality (MR) head-mounted display (HMD), which allows the user to interact with their environment using Holograms whilst engaging their senses throughout, offering an immersive experience. Mixed reality (MR) describes an environment in which real and virtual elements appear to coexist [1]. HMDs such as the HoloLens are becoming increasingly popular in healthcare, particularly in surgical intervention. Medicine is a rapidly evolving science, and the presence of innovation is vital to the development of effective mechanisms to treating and managing disease, delivering optimal healthcare, and assisting medical professionals with executing tasks. Medical professionals should seek to identify and record problems within their workspace to which solutions can be created.

Currently, there exists a problem within minimally invasive surgery, namely that surgeons often operate on patients with a misalignment between their line of vision and hand placement due to monitor positions. Unfortunately, this position is commonly observed amongst various surgeons in many hospitals today. Studies have shown that a disrupted visual-motor axis during surgery can lead to a plethora of problems including declined ergonomics and surgical performance, spatial disorientation, and increased risk of iatrogenic injuries [2]. Although there has been considerable literature concerning AR technologies, with studies describing a similar application of several HMDs, there are currently no published data evaluating the HoloLens as an endoscopic monitor in any surgical speciality. Studies which have been described suffered significant limitations due to the restricted technology of those devices utilised, including loss of spatial awareness, frequent spatial disorientation, extensive cabling, poor battery life and device discomfort [3–11].

The primary aim of this study is to assess the effectiveness of the HoloLens as an endoscopic monitor during minimally invasive surgery—where effectiveness is defined as non-inferior performance-related outcomes, if not superior to the outcomes with a conventional monitor. The secondary aims are to assess the feasibility of introducing the HoloLens in a clinical setting, the ability of the HoloLens to display radiographic imaging and the endoscopic view simultaneously, and to explore the logistical factors of wearing the HoloLens during surgery.

## Materials and methods

### Study design and participants

**Fig. 1:**
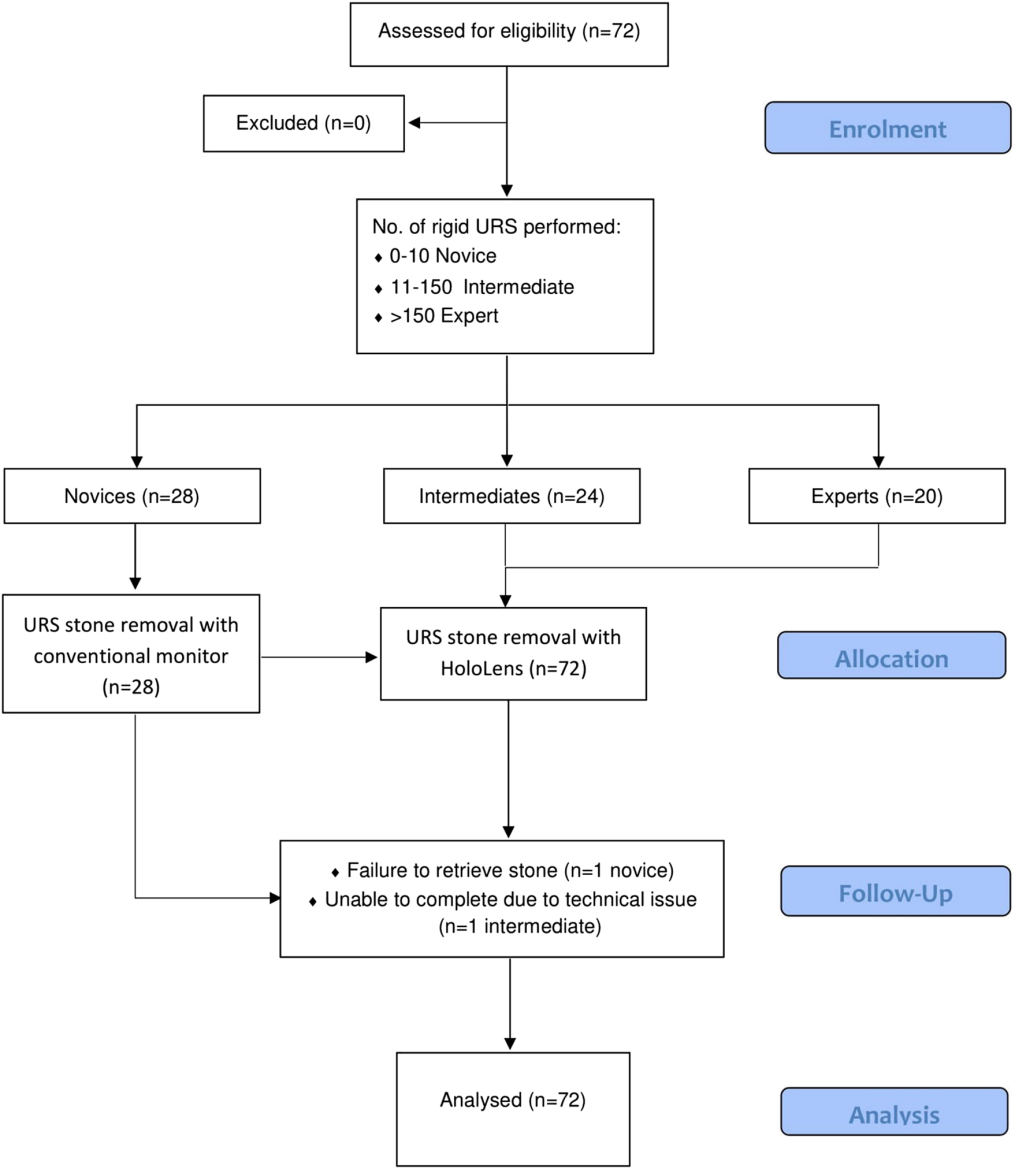
Study design flow chart

This prospective, observational and comparative study recruited 72 participants including novice medical students, urological trainees and specialists. The demographics of the trainees and specialists were highly variable in age and nationality. All participants were classified into different expertise levels based on the number of semi-rigid ureteroscopy procedures previously performed (study design flow chart Fig. 1), thus the following taxonomy was devised: novices 0–10 (*n* = 28); intermediates 11–150 (*n* = 24); experts > 150 (*n* = 20). Participants were eligible for inclusion if they were either a medical student, urological trainee or a specialist. Previous knowledge of performing a ureteroscopy or operating the HoloLens was not necessary. Ethical approval was not required for this study.

### Study process

**Fig. 2:**
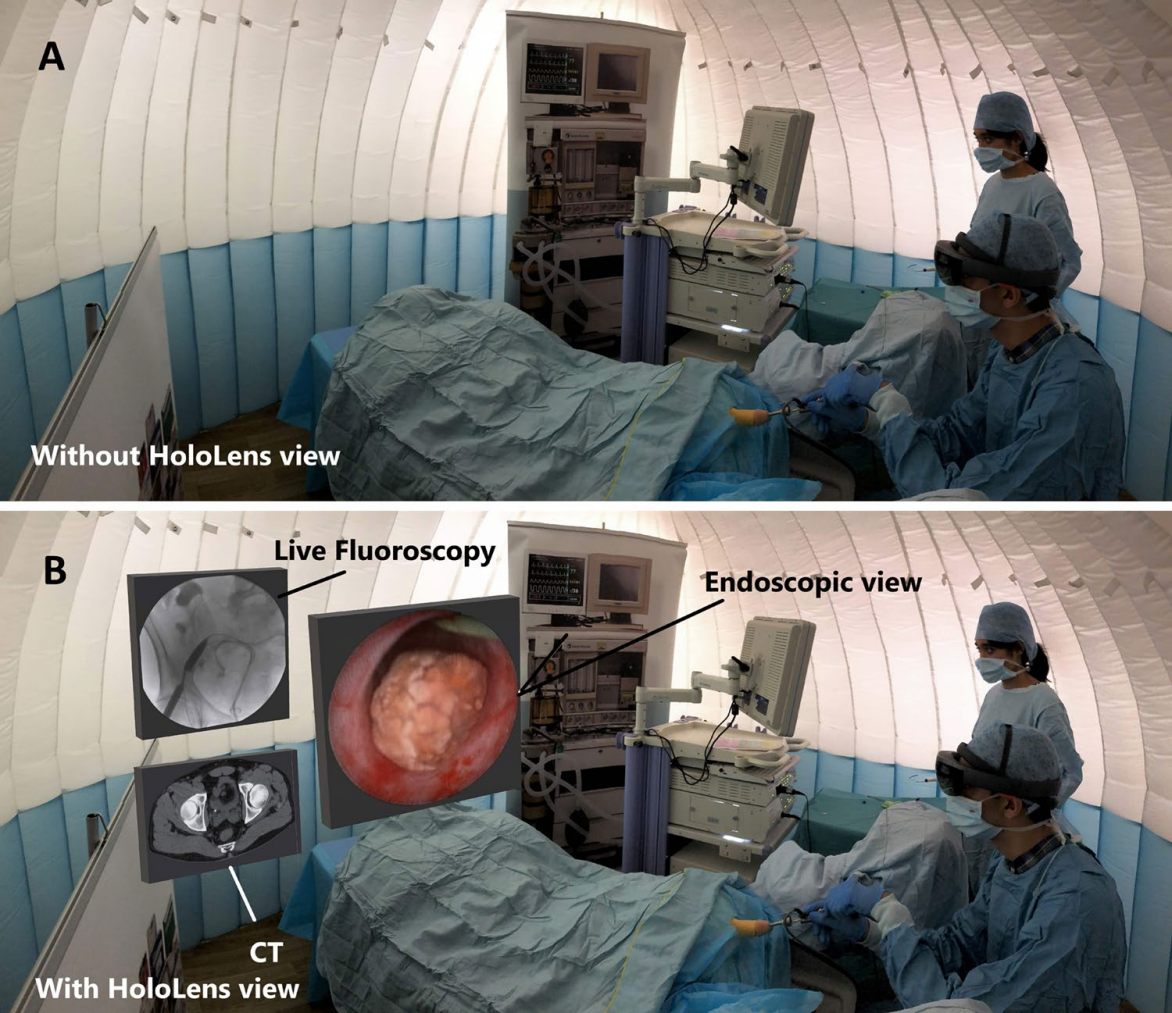
Participants utilising the HoloLens to perform ureteroscopy within a Full Immersion Simulation environment. Image **A:** the view of the user to an outside when the HoloLens is worn, and **B**: a simulated view of what the operator wearing the HoloLens sees

Following recruitment, training material in the format of communication, images and videos were shared with all participants. Novices were taught how to perform ureteroscopy and were allowed to practice the procedure three times prior to the assessments. All participants were taught how to operate the HoloLens using the main gestures and allowed a 15-min practice session. Novices were initially assessed with a conventional monitor positioned at 1 m and an angle of roughly 30° from the participant, followed by an assessment with the HoloLens as the endoscopic monitor (Fig. 2). Both assessments were then repeated to eliminate the practice effect; overall novices performed a total of four procedures. The intermediate and expert groups were not assessed with the conventional monitor due to time constraints, thus a direct comparison with the HoloLens could not be made for these groups. Data from these groups were supplementary and used to correlate performance between the expertise levels.

Participants performed a mid-ureteric stone removal using a basket on two different commonly utilised urological simulators [12]: The Uro-Scopic Trainer (Limbs and Things, Bristol, UK), and the Endo-Uro Trainer (Samed, Dresden, Germany). Training occurred within a previously validated Full Immersion Simulation “Igloo” environment, a concept which aims to create a realistic operating environment at a relatively small cost to teach and assess technical and non-technical skills [13, 14]. Within the inflatable “Igloo” environment, was placed an endoscopy stack, a trolley for the equipment, posters to represent anaesthesia machinery, and a table with a patient doll simulating the operating table. The surgical team inside the “igloo” included the participant and actors who covered the roles of an anaesthetist, a floating nurse, and a scrub nurse.

During training and assessment, real operative equipment were utilised to simulate the realism and applicability of the training. Candidates were required to read a patient scenario provided to ensure adequate preparation. Prior to commencing the procedure, candidates were asked to complete the World Health Organisation (WHO) surgical safety checklist. The HoloLens was calibrated for each participant at the start to provide an optimal view and ensure standardisation.

### Outcome measures

Participant demographics, clinical experience and previous HMD exposure were collected at baseline, using an online survey questionnaire (SurveyMonkey^®^). The primary outcomes assessing performance included procedural time and OSATS, a previously- validated global rating scale for ureteroscopy. Primary outcomes were recorded by an external blinded expert endourologist who was trained in using OSATS. This global rating scale includes seven specific domains: (A) respect for tissue, (B) time and motion, (C) instrument handling, (D) handling of the endoscope, (E) flow of the procedure, (F) use of assistants, and (G) knowledge of the procedure. The maximal Likert score in each domain is five, thus permitting a total of 35 points per assessment. Procedural time was defined as the time difference between initial entry to the external urethral orifice and exit from the orifice with a stone.

Upon completion of the study, feedback questionnaires enquiring about the HoloLens and the symptoms during use were completed using Likert scales and qualitative fields. Participants were asked to describe any symptoms they experienced whilst using the device and rated the severity from 0 to 3, representing “no symptom” to “severe symptom”, respectively.

### Statistical analysis

Qualitative and quantitative data were tabulated, and statistical analysis was performed using PRISM GraphPad version 7.04. Parametric analysis utilising independent t-tests were performed for simple comparisons between modalities in equally distributed data, such as procedural time and OSATS. The Kruskal–Wallis one-way analysis of variance test was conducted to assess variance between the groups of expertise (i.e. novice, intermediates, and experts). A *P* value of < 0.05 was defined to be statistically significant in all tests undertaken.

## Results

All participants enroled in the study completed the tasks provided, with the exception of a novice and intermediate who were unable to retrieve the stone or experienced battery failure during the procedure. In addition, one intermediate participant did not complete the final feedback form.

### Procedural times

**Fig. 3:**
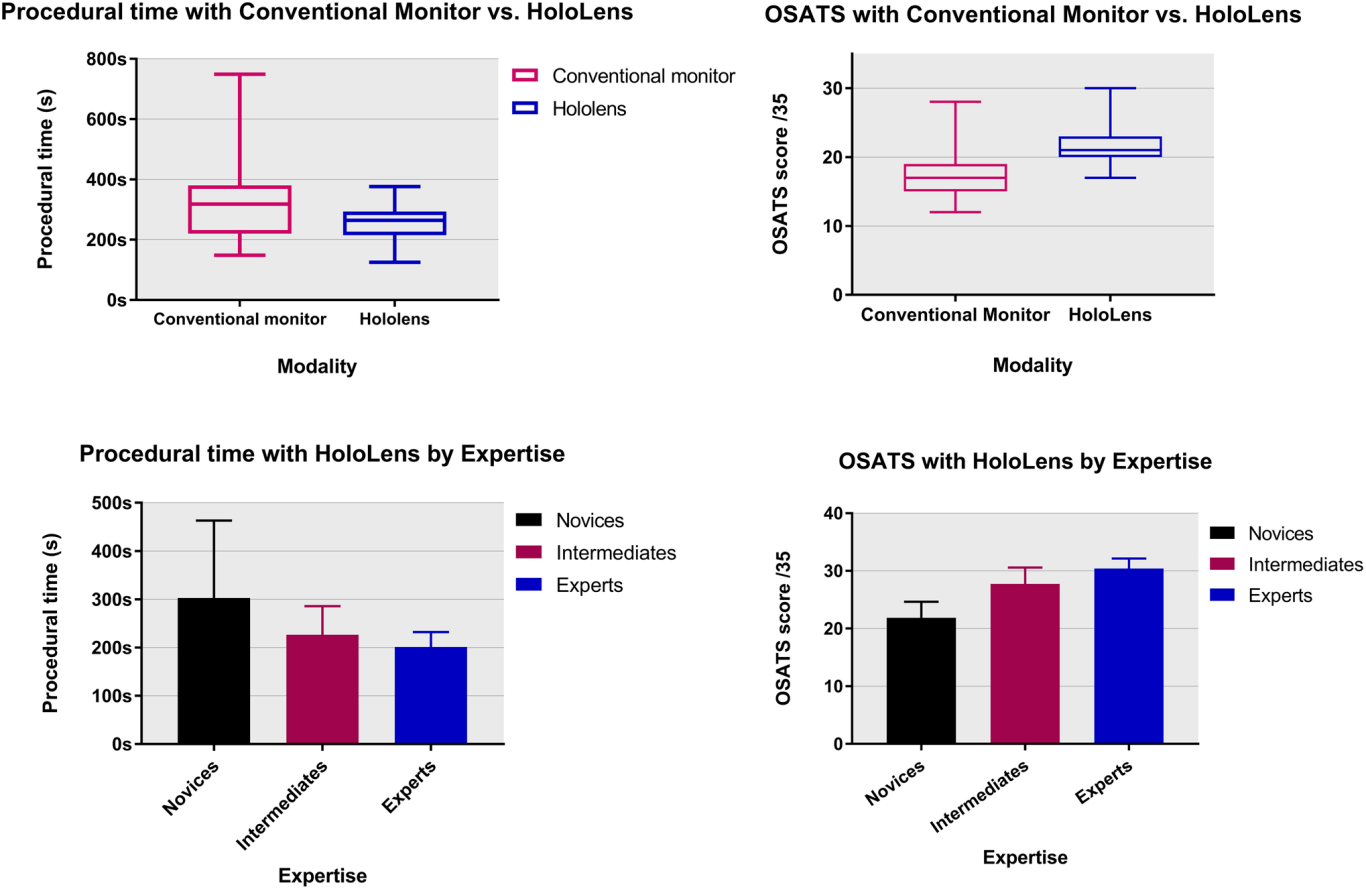
Performance-related outcomes: procedural times (left) and OSATS (right)

The first procedural times with the HoloLens were compared to those of the conventional monitor. Procedural times were shorter on average with the HoloLens compared with a conventional monitor (mean duration (s), 258 vs. 331; absolute difference, − 73 s; 95% CI − 115 to − 30; *P* = 0.0011) (Fig. 3). A Kruskal–Wallis test showed that there was a statistically significant difference in procedural times between the expertise groups (*P* < 0.0001), with a mean rank procedural time of 50.5 for novices, 32.3 for intermediates and 22.0 for experts.

### Performance evaluation scores

OSATS scores with the conventional monitor and HoloLens were compared in the novice group and showed participants scored higher on average using the HoloLens compared with the conventional monitor (17.3 vs. 21.4; absolute difference, 4.1 points; 95% CI 2.9 to 5.3; *P* < 0.0001) (Fig. 3). A Kruskal–Wallis test showed that there was a statistically significant difference in OSATS between the expertise groups (*P* < 0.0001), with a mean rank OSATS of 16.5 for novices, 43.1 for intermediates and 56.6 for experts.

### Feasibility and acceptability

#### Transferability

Feedback from the survey (Table 1) demonstrated that 95% of participants agreed or strongly agreed that the HoloLens will have a role within surgical practice and is feasible to be introduced into the clinical setting (mean rating of 4.4/5, 95% CI 4.2–4.5). Furthermore, 97% of participants agreed or strongly agreed that the HoloLens will have a role in surgical education (mean rating of 4.6/5; 95% CI 4.5–4.8). When asked about the usefulness of the CT images in the HoloLens during the procedure, 77% of participants agreed or strongly agreed that it was a useful feature (mean rating of 4.0/5; 95% CI 3.8–4.2). Feedback regarding image quality, image lag, multitasking ability, comfort, sterility, practicality, and spatial awareness all scored a mean Likert rating of ≥ 4/5 (Table 1).

**Table 1:** LIKERT Feedback from participants regarding the HoloLens

	Rating (1 = strongly disagree, 5 = strongly agree)	
	*M*	SD
Image quality	4.3	0.7
Image lag	4.4	0.7
Multitasking	4.4	0.6
Comfort	4	0.9
Sterility	4.1	0.8
Practicality	4.2	0.7
Spatial awareness	4.2	0.7
The HoloLens was useful in this procedure	4.2	0.7
The HoloLens is not distracting	4.1	0.8
I found the CT images in HoloLens useful	4	0.9
I found the procedural instructions in HoloLens useful	3.8	0.9
I feel the HoloLens will improve patient care during Surgery	4.1	0.8
I feel the HoloLens will have a role within Surgical Practice	4.4	0.6
I feel the HoloLens will have a role within Surgical Education	4.6	0.6
I would like to use the HoloLens in this procedure again	4.5	0.7
How feasible is introducing the HoloLens into simulation programs or clinical practice?	4.4	0.6
The HoloLens has educational value within a simulation training course	4.6	0.5

#### Ease of use

The majority of participants (90%) did not experience any symptoms, whilst the minority who did, all described it to be mild in severity. The most frequent complaints reported were eye fatigue (16%) and neck strain (15%) followed by headaches (5%) and dizziness/nausea (3%). None of the participants experienced any moderate or severe symptoms whilst using the HoloLens.

## Discussion

Effective OR setup remains a challenge in the current setting of minimally invasive surgery. Evidence from ergonomic studies suggests that placement of the endoscopic monitor in alignment with the performing surgeon’s forearm is far more effective in terms of performance, comfort and safety [15]. The guidelines on optimal ergonomics by van Det et al. [15] suggest that the monitor should be placed directly in front of the surgeon, with a maximal angle of 15° in the horizontal plane and approximately 15° downward in the sagittal plane. Viewing distance is highly dependent on monitor size and should be far enough to avoid extensive accommodation of the eyes, whilst remaining close enough to avoid staring and loss of detail, thus emphasising the importance of individualised monitor positioning, which is often overlooked in minimally invasive surgical practice today.

According to an international questionnaire survey [16] conducted amongst 282 surgeons regarding ergonomic factors of minimally invasive surgery, 74% of surgeons reported neck discomfort due to a bad monitor position. Overall 88% agreed that they experience muscle fatigue due to the static posture. Around 80% of surgeons reported a degree of pain in either the neck, shoulder, or lower back regions. When asked about monitor positioning, 36% of the surgeons stated that they would prefer a different position to their current setup [16]. This may be precipitated by theatre equipment in the OR such as the operating table and fluoroscopy c-arm, which may prevent the monitor from being positioned appropriately.

Since development, HMDs have evolved from being heavy, obstructive, and wired devices to become light, see-through, and wireless [3]. The HoloLens offers more immersive technology compared to previous HMD generations and may address the issues of hindered non-technical skills, registration difficulties, portability, sterility and several other factors. In addition, the HoloLens enables the user to visualise multiple holograms simultaneously, allowing integration of other important medical information.

This is the first study to propose an application of the HoloLens as an endoscopy screen, which can be adjusted in size and position to accommodate the surgeon’s preference. Our results demonstrated that in the novice group, all parameters of performance improved with use of the HoloLens compared to conventional monitors. Based on the aims and defined criteria of this feasibility study, the HoloLens proved to be equally as effective as conventional monitors. However, although a statistical significance was found, it may not necessarily correlate with a clinical significance.

This study had several limitations as it was conducted in two different centres, including heterogeneity in settings and training. Consequently, the variability in the equipment utilised may have been a factor with the more advanced systems and monitors providing a better video resolution. Due to logistical factors, intermediates and experts did not receive equal training as novices, which may have impacted their performance and feedback on the device. Finally, there may have been some identity bias due to lack of complete randomisation, lack of blinding and anonymity of the participants.

There were also several limitations to operating the HoloLens. Contrary to the way the device is widely marketed, the HoloLens has a restricted projection size, thus affecting the immersive experience. Upon turning the head for instance, the borders of the screen may cut off, therefore limiting the number of screens that can be supported simultaneously. In addition, some participants also had difficulty utilising the head strap and visor adjustments effectively, which may have contributed to the reported rates of neck strain. The HoloLens may propose further symptomatic problems in lengthier procedures, particularly exacerbating neck strain. Moreover, battery failure occurred mid-way through procedure in the case of one participant and consequently prevented completion of the study. It is therefore crucial that future editions of this device or alternative mixed-reality HMDs are to be made lighter with good battery life to facilitate lengthier and more complex procedures in the OR.

## Conclusion

Our study demonstrates that the device facilitated improved outcomes of performance and was widely accepted as a surgical visual aid by the study participants. HoloLens represents a feasible alternative to conventional endoscopic monitors, possibly by aligning the surgeon’s visual-motor axis. The device is operated using gestures, and thus sterility is not compromised and can support safe practice. However, clinicians should undergo comprehensive training to ensure safe practice in the OR. Although an improvement in the performance outcomes with statistical significance was shown, it may not necessarily correlate with a clinical significance. Further evaluation in the clinical setting is underway.

## Abbreviations

HL: HoloLens
HMD: Head-mounted display
AR: Augmented reality
MR: Mixed reality
VR: Virtual reality
PCNL: Percutaneous nephrolithotomy
OR: Operating room
